# High-normal serum bilirubin decreased the risk of lower limb atherosclerosis in type 2 diabetes: a real-world study

**DOI:** 10.1186/s13098-023-01088-9

**Published:** 2023-05-19

**Authors:** Cui-Chun Zhao, Jun-Wei Wang, Ming-Yun Chen, Jiang-Feng Ke, Mei-Fang Li, Lian-Xi Li

**Affiliations:** 1 Department of Endocrinology & Metabolism, Shanghai Sixth People’s Hospital Affiliated to Shanghai Jiao Tong University School of Medicine, Shanghai Clinical Medical Center of Diabetes, Shanghai Key Clinical Center of Metabolic Diseases, Shanghai Institute for Diabetes, Shanghai Key Laboratory of Diabetes, Shanghai, China; 2 Department of VIP, Shanghai Sixth People’s Hospital Affiliated to Shanghai Jiao Tong University School of Medicine, Shanghai, China; 3 Department of Endocrinology, The First Affiliated Hospital of Fujian Medical University, Fujian, China; 4 Department of Emergency, Shanghai Sixth People’s Hospital Affiliated to Shanghai Jiao Tong University School of Medicine, Shanghai, China

**Keywords:** Serum total bilirubin, Serum unconjugated bilirubin, Serum conjugated bilirubin, Lower limb atherosclerosis, Lower limb plaque, Lower limb stenosis, Type 2 diabetes mellitus

## Abstract

**Background:**

Bilirubin has been found to protect against overt atherosclerotic diseases, but to date, few studies have investigated the effects of bilirubin especially within the normal range on lower limb atherosclerosis. Therefore, we aimed to assess the associations of bilirubin within normal limits including total bilirubin (TB), conjugated bilirubin (CB) and unconjugated bilirubin (UCB) with lower limb atherosclerosis in Chinese patients with type 2 diabetes mellitus (T2DM).

**Methods:**

7284 T2DM patients with normal levels of serum bilirubin were included in this cross-sectional, real-world study. Patients were divided into quintiles by TB levels (< 8.7, 8.7-10.19, 10.20-11.99, 12-13.99, > 13.99 µmol/L). Lower limb ultrasonography was conducted to detect lower limb plaque and stenosis. The association between serum bilirubin and lower limb atherosclerosis was explored by multiple logistic regression.

**Results:**

A remarkable decrease in the prevalence of lower limb plaque (77.5, 75.3, 70.7, 71.7 and 67.9%) and stenosis (21.1, 17.2, 13.3, 13.0 and 12.0%) was observed across the TB quintiles. Multivariable regression analysis showed that serum TB levels were negatively correlated with higher risks of lower limb plaque and stenosis, both as a continuous variable [OR (95%CI): 0.870 (0.784–0.964), *p* = 0.008 for plaque; and 0.835 (0.737–0.946), *p* = 0.005 for stenosis] and as categorized in quintiles (*p* = 0.015 and 0.016 for plaque and stenosis). Interestingly, serum CB levels were only negatively correlated with lower limb stenosis [OR (95%CI): 0.767 (0.685–0.858), *p* < 0.001], whereas serum UCB levels were only negatively associated with lower limb plaque [ OR (95%CI): 0.864 (0.784–0.952), *p* = 0.003] after a fully-adjusted analysis. Furthermore, serum CRP was significantly decreased across the TB quintiles and negatively associated with serum TB (r = -0.107, *p* < 0.001), CB (r = -0.054, *p* < 0.001), and UCB (r = -0.103, *p* < 0.001).

**Conclusions:**

High-normal serum bilirubin levels were independently and significantly related to reduced risks of lower limb atherosclerosis in T2DM patients. Furthermore, serum bilirubin levels including TB, CB and UCB were inversely correlated with CRP. These results suggested that higher-normal serum bilirubin may exhibit an anti-inflammatory and protective effect against lower limb atherosclerotic progression in T2DM subjects.

**Supplementary Information:**

The online version contains supplementary material available at 10.1186/s13098-023-01088-9.

## Background

Atherosclerosis, as the leading cause of peripheral arterial disease (PAD) and non-traumatic lower-extremity amputation (LEA), is one of the most severe complications of diabetes [1, 2]. Poor glycemic control accelerates the development of atherosclerosis in patients with diabetes. It is documented that an individual with diabetes suffered at least 8 times of risk to undergo a nontraumatic amputation compared with a non-diabetic individual [2, 3]. Furthermore, diabetes-related lower limb atherosclerosis and its complications not only significantly reduce life quality and expectancy of diabetic patients, but also place huge burdens on health care systems and societies [4, 5]. Therefore, it is urgently required to further deepen understandings on potential risk factors of the lower limb atherosclerosis in type 2 diabetes mellitus (T2DM) to prevent these adverse conditions.

Bilirubin, once considered just a useless end product of heme metabolism, has been found to possess powerful antioxidant and neuroprotective properties in recent decades [6, 7]. In recent years, bilirubin has attracted attentions as a physiological regulator of oxidative stress in diabetic patients [8]. A negative relationship has been observed between serum bilirubin and surrogate markers of PAD such as ankle brachial index, arterial stiffness in different populations [9, 10]. Additionally, several observational studies have shown that serum bilirubin is negatively correlated with diabetic nephropathy, retinopathy, and neuropathy [11–13]. However, up to now, only one study of 464 patients with familial dyslipidemia evaluated the association of serum total bilirubin (TB) with lower limb atherosclerosis and demonstrated that a negative correlation between serum TB and femoral plaque thickness was only existed in the subjects with familial combined hyperlipidemia [14].

Furthermore, above-mentioned clinical studies mainly focused on TB and did not distinguish conjugated bilirubin (CB), also known as direct bilirubin (D-BIL) and unconjugated bilirubin (UCB), also known as indirect bilirubin (I-BIL) from TB. Little is known about the role of bilirubin on lower limb atherosclerosis in T2DM patients, despite evidence in favor of the hypothesis that serum bilirubin protects against overt cardiovascular disease [15, 16]. Therefore, in this study, we explored the feasibility of serum bilirubin levels within the normal range, including TB, CB and UCB, as indicators to evaluate the risk of lower limb atherosclerosis including lower limb plaque and stenosis in Chinese T2DM patients.

## Materials and methods

### Subjects and study design

This was a cross-sectional, real-world study based on the data of 11,805 T2DM patients in our recent study [17]. In addition to the exclusion criteria in that study [17], we also excluded the patients without data of lower limb ultrasound examination in this study. Finally, the remaining 7284 T2DM patients took part in our present study. This study was approved by the Ethics Committee of Shanghai Sixth People’s Hospital Affiliated to Shanghai Jiao Tong University School of Medicine (approval number: 2018-KY-018(K)) and written informed consent was obtained from each participant.

### Physical examination and laboratory measurements

Information on social habits such as smoking, alcohol use, as well as medical and medication histories were collected at admission and anthropometric measurements including blood pressure, weight, height, waist circumference, and hip circumference were conducted based on our recent studies [18–20]. Body mass index (BMI) was obtained as weight (kg) divided by the square of height (m), and the waist-to-hip ratio (WHR) was computed as waist circumference divided by hip circumference.

Fasting and 2-hour postprandial blood samples were collected on the second day of admission for laboratory analysis. Specifically, serum TB and CB levels were measured using LABOSPECT 008AS autoanalyzer (Hitachi, Tokyo) and serum UCB level was obtained by TB concentration minus CB concentration as described in our current study [17]. Other laboratory parameters including blood glucose, glycosylated hemoglobin A1c (HbA1c), insulin, C-peptide, lipid profiles, liver and renal function, C-reactive protein (CRP) as well as urine tests were measured as described in our previous studies [18–20]. The 24 h urinary albumin excretion (UAE) was defined as the average value of three separate early morning urine samples during hospitalization. The homeostasis model assessment index of insulin resistance (HOMA-IR) was computed as fasting plasma insulin (mU/l) × fasting plasma glucose (mmol/l)/22.5 [21]. The homeostasis model assessment for insulin resistance (HOMA2-IR) was made by the HOMA calculator version 2.2.3 and the estimated glomerular filtration rate (eGFR) was computed according to our previous method [18–20].

### Doppler ultrasonography examinations of lower limb

The lower limb ultrasonographic examinations were conducted according to the standard protocols which were well performed in our previous studies [22–24]. Briefly, after the patients remained in the supine position for 5 min, the 5-13-MHz linear array transducer was successively placed on seven locations of bilateral lower limb arteries including common femoral artery, profunda femoris artery, superficial femoral artery, popliteal artery, anterior tibial artery, posterior tibial artery, and peroneal artery to measure and record atherosclerotic plaque and stenosis using an Acuson Sequoia 512 scanner [23, 24].

### Diagnostic criteria

The definitions of smoking status, alcohol use, obesity and hypertension had been described in detail previously by our team [23, 24]. The definitions of lower limb plaque and stenosis had also been well-written in our early studies [23, 24]. Briefly, lower limb plaque was identified as the presence of atherosclerotic plaque in any of the above-mentioned lower limb artery segments [23, 24]. Lower limb stenosis was diagnosed as any degree of narrowing of the lower limb arteries caused by plaque [23, 24].

### Statistical analyses

Statistical analyses were made using SPSS version 15.0 and figures were performed by GraphPad Prism 7.0. The Kolmogorov-Smirnov Test was adopted to examine the normal distribution for continuous variables. Data with normal distribution were represented as mean ± S.D., and those with non-normal distribution were expressed as median and interquartile ranges (25-75%). One-way ANOVA or Kruskal-Wallis H test was utilized to compare continuous variables among groups. Categorical variables given as absolute numbers and percentages were compared by Chi-square test. Spearman’s correlation analysis was used to evaluate the interrelationship between serum bilirubin and CRP. Binary logistic regression analyses were applied to assess the correlations of serum bilirubin levels and quintiles with lower limb atherosclerosis. *P* < 0.05 was regarded as statistically significant.

## Results

### Characteristics of the study subjects according to TB quintiles

All the study subjects were divided into TB quintiles with cutoffs of < 8.7, 8.7-10.19, 10.20-11.99, 12-13.99, > 13.99 µmol/L based on the TB concentration. The demographic data and clinical characteristics of the studied subjects stratified by TB quintiles are manifested in Table 1. Individuals in the higher TB quintiles were more likely to be male and younger, and had significantly higher levels of alanine aminotransferase (ALT), aspartate aminotransferase (AST), γ-glutamyl transpeptidase (γ-GT), as well as shorter duration of diabetes (DD), lower UAE even after adjusting for age and/or sex. Remarkable differences were also observed in smoking, the use of lipid-lowering drugs (LLDs) and antiplatelet agents (APAs) and insulin or insulin analogues (IIAs), fasting plasma glucose (FPG), 2-h postprandial plasma glucose (2 h PPG), HbA1c, 2 h insulin (2 h ins), HOMA-IR, 2-h postprandial C-peptide (2 h PCP), HOMA2-IR, total triglycerides (TTG), low density lipoprotein-cholesterol (LDL-C), Lipoprotein a (Lp(a)), creatinine (Cr), serum uric acid (SUA), and eGFR among the five groups after adjusting for age and sex (all *p* < 0.05).

**Table 1:** Characteristics of the study subjects according to TB quintiles

Variables	Q1 (n = 1432)	Q2 (n = 1481)	Q3 (n = 1215)	Q4 (n = 1417)	Q5 (n = 1739)	p value	*p value
TB (umol/l)	< 8.70	8.70-10.19	10.20-11.99	12.00-13.99	> 13.99	—	—
Male (n, %)	592(41.3%)	687(46.4%)	609(50.1%)	736(51.9%)	1102(63.4%)	< 0.001	< 0.001
Age (years)	62 ± 12	61 ± 12	60 ± 12	60 ± 12	58 ± 12	< 0.001	< 0.001
^a^DD (months)	120(60–180)	108(48–168)	96(36–168)	84(24–138)	84(24–132)	< 0.001	< 0.001
Hypertension (n, %)	850(59.4%)	831(56.1%)	684(56.3%)	763(53.8%)	903(51.9%)	0.001	0.255
Obesity (n, %)	653(45.6%)	669(45.2%)	566(46.6%)	662(46.7%)	804(46.2%)	0.911	0.942
Smoking (n, %)	372(26%)	400(27%)	338(27.8%)	387(27.3%)	561(32.3%)	0.001	< 0.001
Alcohol (n, %)	171(11.9%)	188(12.7%)	183(15.1%)	215(15.2%)	339(19.5%)	< 0.001	0.809
LLD (n, %)	667(46.6%)	591(39.9%)	501(41.2%)	541(38.2%)	609(35%)	< 0.001	< 0.001
AHAs (n, %)	802(56%)	763(51.5%)	634(52.2%)	706(49.8%)	836(48.1%)	< 0.001	0.114
APAs (n, %)	797(55.7%)	784(52.9%)	661(54.4%)	749(52.9%)	851(48.9%)	0.003	0.020
IIAs (n, %)	1073(74.9%)	1059(71.5%)	820(67.5%)	937(66.1%)	1166(67.1%)	< 0.001	< 0.001
SBP (mmHg)	134 ± 17	133 ± 18	133 ± 17	133 ± 17	132 ± 17	0.201	0.869
DBP (mmHg)	79 ± 9	79 ± 9	80 ± 10	80 ± 9	81 ± 10	< 0.001	0.120
WC (cm)	89.5 ± 10.7	89.7 ± 10.4	89.7 ± 10.4	89.7 ± 10.3	90.2 ± 10.3	0.574	0.063
WHR	0.92 ± 0.07	0.92 ± 0.07	0.92 ± 0.07	0.92 ± 0.06	0.92 ± 0.06	0.923	0.369
BMI (kg/m^2^)	24.87 ± 3.54	24.81 ± 3.53	24.95 ± 3.48	24.87 ± 3.43	24.94 ± 3.47	0.824	0.895
^a^FPG (mmol/l)	7.29(5.89–9.46)	7.70(6.21–9.67)	7.50(6.21–9.60)	7.82(6.29–9.71)	8.34(6.58–10.30)	< 0.001	< 0.001
^a^2h PPG (mmol/l)	12.36(9.50-15.59)	13.14(10.01–16.60)	13.03(10.09–16.36)	13.53(10.33–16.63)	14.28(11.09–17.62)	< 0.001	< 0.001
HbA1c (%)	8.7 ± 2.2	9.0 ± 2.2	8.8 ± 2.2	8.9 ± 2.2	9.0 ± 2.1	0.001	0.004
^a^Fins (uU/ml)	1.87(1.18–2.82)	1.79(1.08–2.61)	1.83(1.17–2.54)	1.77(1.15–2.60)	1.82(1.24–2.58)	0.136	0.089
^a^2h ins (uU/ml)	3.88(2.29–6.04)	3.91(2.09–6.03)	4.13(2.42–6.12)	4.22(2.43–6.21)	4.08(2.51–6.06)	< 0.001	< 0.001
aHOMA-IR	4.56(2.65–8.53)	4.65(2.73–8.50)	4.27(2.43–7.31)	4.34(2.61–7.03)	4.45(2.77–7.28)	0.001	0.001
^a^FCP (ng/mL)	1.87(1.18–2.82)	1.79(1.08–2.61)	1.83(1.17–2.54)	1.77(1.15–2.60)	1.82(1.24–2.58)	0.136	0.089
^a^2h PCP (ng/mL)	3.88(2.29–6.04)	3.91(2.09–6.03)	4.13(2.42–6.12)	4.22(2.43–6.21)	4.08(2.51–6.06)	0.034	0.011
^a^HOMA2-IR	1.58(0.99–2.43)	1.54(0.91–2.27)	1.59(1.00-2.19)	1.53(0.99–2.24)	1.60(1.05–2.28)	0.085	0.045
^a^TG (mmol/l)	1.52(1.05–2.38)	1.47(1.04–2.16)	1.40(0.99–2.08)	1.39(0.97–2.10)	1.51(1.01–2.18)	< 0.001	< 0.001
TC (mmol/l)	4.86 ± 1.32	4.84 ± 1.15	4.81 ± 1.06	4.80 ± 1.03	4.85 ± 1.11	0.589	0.241
HDL-C (mmol/l)	1.08 ± 0.29	1.13 ± 0.30	1.15 ± 0.31	1.15 ± 0.31	1.16 ± 0.33	< 0.001	0.487
LDL-C (mmol/l)	2.97 ± 0.98	3.06 ± 0.94	3.10 ± 0.93	3.09 ± 0.87	3.14 ± 0.95	< 0.001	< 0.001
^a^Lp (a)	10.8(5.6–22.6)	10.9(6.6–21.4)	10.5(5.7–21.6)	10.7(5.7–21.5)	10.3(5.2–19.9)	0.030	0.004
^a^ALT (U/l)	17(12–25)	18(13–26)	19(13–29)	20(14–31)	21(15–32)	< 0.001	< 0.001
^a^AST (U/l)	18(15–23)	18(15–23)	19(13–24)	20(16–25)	20(16–26)	< 0.001	< 0.001
^a^γ-GT (U/l)	22(16–33)	22(16–34)	24(17–35)	25(17–39)	26(18–41)	< 0.001	< 0.001
^a^Cr (µmol/l)	66(54–83)	65(54–79)	65(54–78)	65(54–77)	67(56–78)	0.004	0.005
^a^SUA (µmol/l)	317(264–384)	308(254–370)	315(264–376)	310(256–373)	313(258–373)	0.036	< 0.001
^a^UAE (mg/24 h)	15.39(7.46–76.53)	12.00(6.95–38.30)	11.68(7.07–30.75)	11.17(6.99–24.91)	10.80(6.61–25.19)	< 0.001	< 0.001
^a^eGFR (ml/min/1.73 m^2^)	106(81–130)	109(89–132)	110(91–134)	111(92–133)	111(94–133)	< 0.001	< 0.001

Abbreviations: TB, total bilirubin; DD, duration of diabetes; LLDs, lipid-lowering drugs; AHAs, antihypertensive agents; APAs, anti-platelet agents; IIAs, insulin or insulin analogues; SBP, systolic blood pressure; DBP, diastolic blood pressure; WC, waist circumstance; WHR, waist–hip ratio; BMI, body mass index; FPG, Fasting plasma glucose; 2 h PPG, 2-hour postprandial plasma glucose; HbA1c, glycosylated hemoglobin A1c; Fins, fasting insulin; 2hins, 2 h insulin; HOMA-IR, the Homeostasis Model Assessment Indexes-Insulin Resistance; FCP, fasting C-peptide; 2 h PCP, 2-hour postprandial C-peptide; TTG, total triglycerides; TC, total cholesterol; HDL-C, high density lipoprotein-cholesterol; LDL-C, low density lipoprotein-cholesterol; Lp(a), Lipoprotein a; ALT, aspartate aminotransferase; AST, aspartate aminotransferase; γ-GT, γ-glutamyl transpeptidase; Cr, creatinine; SUA, serum uric acid; UAE, urinary albumin excretion; eGFR, estimated glomerular filtration rate; CRP, C-reactive protein

Values are expressed as the mean ± S.D, or median with interquartile range, or percentages. ^a^ Non-normal distribution of continuous variables

P-value: The p-values were not adjusted for age for the trend. *P-value: The *p-values were adjusted for age and sex for the trend

### Characteristics of serum bilirubin levels in the study subjects

The characteristics of serum bilirubin including TB, CB and UCB in the study subjects by the stratification of sex, age, and DD were displayed in Supplementary Fig. 1. After controlling for age and DD, men patients had prominently elevated TB, CB and UCB levels compared with women (all *p* < 0.001, Supplementary Fig. 1A, 1D, 1G). A remarkably decreased trend was successively observed in TB, CB and UCB levels with prolonged DD after adjustment for age and sex (all *p* < 0.001 for trend, Supplementary Fig. 1 C, 1 F and 1I). Furthermore, notably decreased TB and CB levels (*p* = 0.032 and 0.001, respectively), but not UCB, were found with increasing age after adjustment for sex and DD (Supplementary Fig. 1B, 1E and 1 H).

### Characteristics of lower limb atherosclerosis in the study subjects

The characteristics of lower limb atherosclerosis in the study subjects stratified by sex, age, and DD were demonstrated in Supplementary Fig. 2. After adjustment for age and DD, male patients had notably higher prevalence of lower limb plaque (74.1% vs. 70.9%) and stenosis (16.7% vs. 13.7%) compared with female (Supplementary Fig. 2A, 2D). A significantly increasing prevalence of lower limb plaque and stenosis was noted with age and DD, respectively (Supplementary Fig. 2B, 2 C and 2E, 2 F).

### Comparisons of lower limb atherosclerosis among the TB quintile groups

**Fig. 1:**
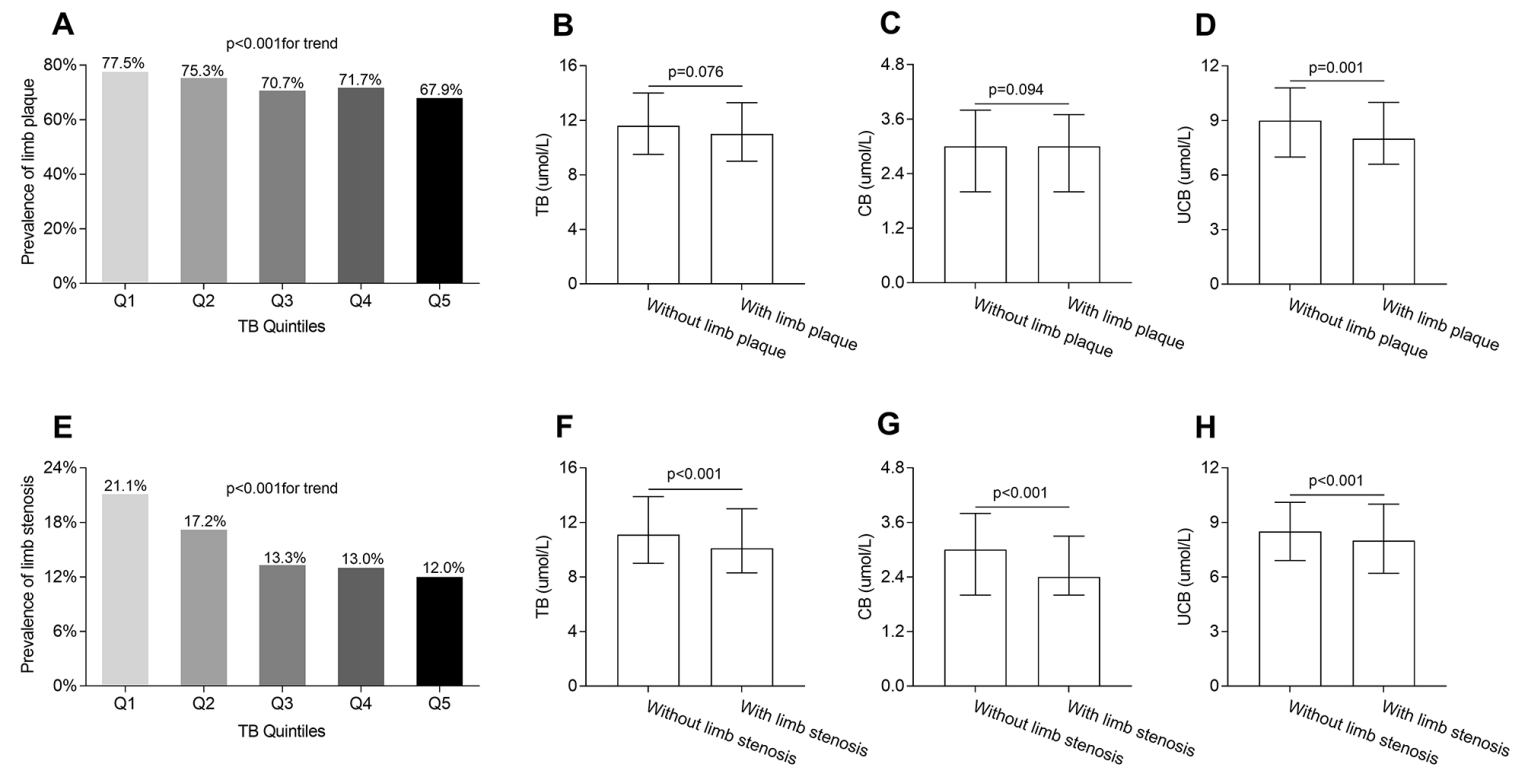
Comparisons of lower limb atherosclerosis among the TB quintile groups. (**A**) Comparison of the prevalence of lower limb plaque among the TB quintile groups after adjusting for age, sex, and DD. (**B**) Comparison of TB levels between the subjects with and without lower limb plaque after controlling for age, sex and DD. (**C**) Comparison of CB levels between the subjects with and without lower limb plaque after controlling for age, sex and DD. (**D**) Comparison of UCB levels between the subjects with and without lower-limb plaque after controlling for age, sex and DD. (**E**) Comparison of the prevalence of lower limb stenosis among the TB quintile groups after adjusting for age, sex, and DD. (**F**) Comparison of TB levels between the subjects with and without lower limb stenosis after controlling for age, sex and DD. (**G**) Comparison of CB levels between the subjects with and without lower limb stenosis after controlling for age, sex and DD. (**H**) Comparison of UCB levels between the subjects with and without lower limb stenosis after controlling for age, sex and DD.

Figure 1 illustrates the comparisons of lower limb atherosclerosis among the TB quintile groups. As shown in Fig. 1, a remarkable decrease in the prevalence of lower limb plaque and stenosis was found across the TB quintiles (lower limb plaque: 77.5, 75.3, 70.7, 71.7 and 67.9%, respectively, *p* < 0.001 for trend; lower limb stenosis: 21.1, 17.2, 13.3, 13.0 and 12.0%, respectively, *p* < 0.001 for trend) (Fig. 1A and E). Additionally, a significantly lower serum UCB concentration, but not TB and CB concentrations, was detected in the patients with lower limb plaque compared with those without lower limb plaque [8.0 (IQR 6.6–10.0) vs. 9.0 (IQR 7.0-10.8) µmol/L, *p* = 0.001] (Fig. 1B C and 1D). However, the levels of TB [10.1 (IQR 8.3–13.0) vs. 11.1 (IQR 9.0-13.9) µmol/L, *p* < 0.001], CB [2.4 (IQR 2.0-3.3) vs. 3.0 (IQR 2.0-3.8) µmol/L, *p* < 0.001] and UCB [8.0 (IQR 6.2–10.0) vs. 8.5 (IQR 6.9–10.1) µmol/L, *p* < 0.001] were all obviously lower in the patients with lower limb stenosis compared with those without lower limb stenosis (Fig. 1F and G H).

### Associations of serum bilirubin levels with lower limb plaque and stenosis

Table 2 displays the associations of serum TB, CB, and UCB levels with lower limb plaque and stenosis. Serum TB levels within the normal limits were negatively correlated with lower limb plaque and stenosis in unadjusted model. Further controlling for various clinical indicators (Model 2–6), serum TB levels remained as an independent risk factor for lower limb plaque [odds ratio (OR) 0.870, 95% confidence interval (CI) 0.784–0.964; *p* = 0.008] and stenosis [OR 0.835, 95% CI 0.737–0.946; *p* = 0.005]. Notably, serum CB levels within the normal limits were only negatively correlated with lower limb stenosis [OR 0.767, 95%CI 0.685–0.858; *p* < 0.001], but not with lower limb plaque; while serum UCB levels within the normal limits were only negatively associated with lower limb plaque [OR 0.864, 95%CI 0.784–0.952; *p* = 0.003], but not with lower limb stenosis, after adjustment for other risk factors.

**Table 2:** Associations of serum bilirubin levels with lower limb atherosclerosis

	TB levels				CB levels				UCB levels			
	OR	95%CI	P value		OR	95%CI	P value		OR	95%CI	P value	
Lower limb plaque												
Model 1	0.802	0.750–0.857	< 0.001		0.923	0.868–0.982	0.011		0.798	0.749–0.850	< 0.001	
Model 2	0.836	0.770–0.906	< 0.001		0.911	0.843–0.984	0.018		0.841	0.779–0.907	< 0.001	
Model 3	0.837	0.772–0.908	< 0.001		0.913	0.844–0.986	0.021		0.841	0.779–0.908	< 0.001	
Model 4	0.852	0.784–0.926	< 0.001		0.940	0.867–1.019	0.132		0.848	0.785–0.918	< 0.001	
Model 5	0.875	0.797–0.960	0.005		0.947	0.865–1.036	0.234		0.871	0.798–0.951	0.002	
Model 6	0.870	0.784–0.964	0.008		0.950	0.855–1.055	0.338		0.864	0.784–0.952	0.003	
**Lower limb stenosis**												
Model 1	0.728	0.673–0.787	< 0.001		0.746	0.692–0.804	< 0.001		0.793	0.736–0.855	< 0.001	
Model 2	0.767	0.700-0.841	< 0.001		0.695	0.636–0.759	< 0.001		0.877	0.804–0.957	0.003	
Model 3	0.789	0.719–0.867	< 0.001		0.706	0.645–0.772	< 0.001		0.901	0.824–0.984	0.021	
Model 4	0.800	0.728–0.879	< 0.001		0.711	0.650–0.779	< 0.001		0.911	0.833–0.996	0.041	
Model 5	0.819	0.736–0.912	< 0.001		0.736	0.666–0.814	< 0.001		0.930	0.840–1.029	0.160	
Model 6	0.835	0.737–0.946	0.005		0.767	0.685–0.858	< 0.001		0.925	0.820–1.043	0.205	

Model 1: unadjusted

Model 2: age, sex, and DD

Model 3: Model 2 + smoking status, alcohol intake, hypertension, and obesity

Model 4: Model 3 + use of APAs, AHAs, LLDs and IIAs

Model 5: Model 4 + SBP, DBP, WC, WHR and BMI

Model 6: Model 5 + ALT, AST, γ-GT, TTG, TC, HDL-C, LDL-C, Lp(a), eGFR, SUA, UAE, FPG, 2 h PPG, HbA1C, FCP, 2 h PCP, Fins, 2hins, and CRP

### Association of serum TB quintiles with lower limb plaque and stenosis

Table 3 demonstrates the associations of serum TB quintiles with lower limb plaque and stenosis. The remarkably inverse association of TB quintiles with lower limb plaque and stenosis were shown in Model 1. The correlation was still significant even after adjusting for other potential confounders (Model 2–6). Accordingly, the fully adjusted analysis showed that compared with the first TB quintile, the risk of lower limb plaque decreased by 32.3%, 23.0%, 22.6% for those from the third to fifth TB quintile, whereas no significance was observed between the second TB quintile and the first TB quintile. And the risk of lower limb stenosis reduced by 30.3%, 33.6%, 33.9%, 29.4% respectively, for those from the second to highest TB quintile when compared with the subjects in the lowest TB quintile.

**Table 3:** Association of serum TB quintiles with lower limb atherosclerosis

	ORs (95%CI)					p value
	Q1	Q2	Q3	Q4	Q5	for trend
Lower limb plaque						
Model 1	1(ref)	0.884(0.745–1.049)	0.700(0.588–0.834)	0.735(0.620–0.871)	0.614(0.523–0.720)	< 0.001
Model 2	1(ref)	0.890(0.723–1.095)	0.667(0.540–0.825)	0.732(0.595–0.901)	0.683(0.560–0.833)	< 0.001
Model 3	1(ref)	0.894(0.726–1.101)	0.668(0.540–0.826)	0.736(0.598–0.905)	0.686(0.563–0.837)	< 0.001
Model 4	1(ref)	0.934(0.755–1.156)	0.680(0.547–0.845)	0.762(0.616–0.942)	0.726(0.592–0.890)	0.001
Model 5	1(ref)	0.955(0.755–1.207)	0.634(0.501–0.803)	0.762(0.602–0.965)	0.797(0.633–1.003)	0.001
Model 6	1(ref)	0.964(0.746–1.246)	0.677(0.522–0.877)	0.770(0.597–0.992)	0.774(0.601–0.997)	0.015
**Lower limb stenosis**						
Model 1	1(ref)	0.778(0.647–0.937)	0.572(0.464–0.704)	0.558(0.457–0.682)	0.511(0.422–0.620)	< 0.001
Model 2	1(ref)	0.775(0.627–0.959)	0.582(0.460–0.737)	0.582(0.463–0.730)	0.600(0.480–0.749)	< 0.001
Model 3	1(ref)	0.780(0.628–0.968)	0.600(0.472–0.762)	0.619(0.491–0.779)	0.627(0.501–0.785)	< 0.001
Model 4	1(ref)	0.790(0.635–0.982)	0.605(0.475–0.770)	0.631(0.500-0.796)	0.652(0.520–0.819)	< 0.001
Model 5	1(ref)	0.783(0.612–1.002)	0.667(0.512–0.869)	0.648(0.498–0.842)	0.718(0.554–0.931)	0.006
Model 6	1(ref)	0.697(0.529–0.918)	0.664(0.496–0.889)	0.661(0.494–0.884)	0.706(0.524–0.953)	0.016

Model 1: unadjusted

Model 2: age, sex, and DD

Model 3: Model 2 + smoking status, alcohol intake, hypertension, and obesity

Model 4: Model 3 + use of APAs, AHAs, LLDs and IIAs

Model 5: Model 4 + SBP, DBP, WC, WHR and BMI

Model 6: Model 5 + ALT, AST, γ-GT, TTG, TC, HDL-C, LDL-C, Lp(a), eGFR, SUA, UAE, FPG, 2 h PPG, HbA1C, FCP, 2 h PCP, Fins, 2hins, and CRP

### Association of serum bilirubin levels with CRP

**Fig. 2:**
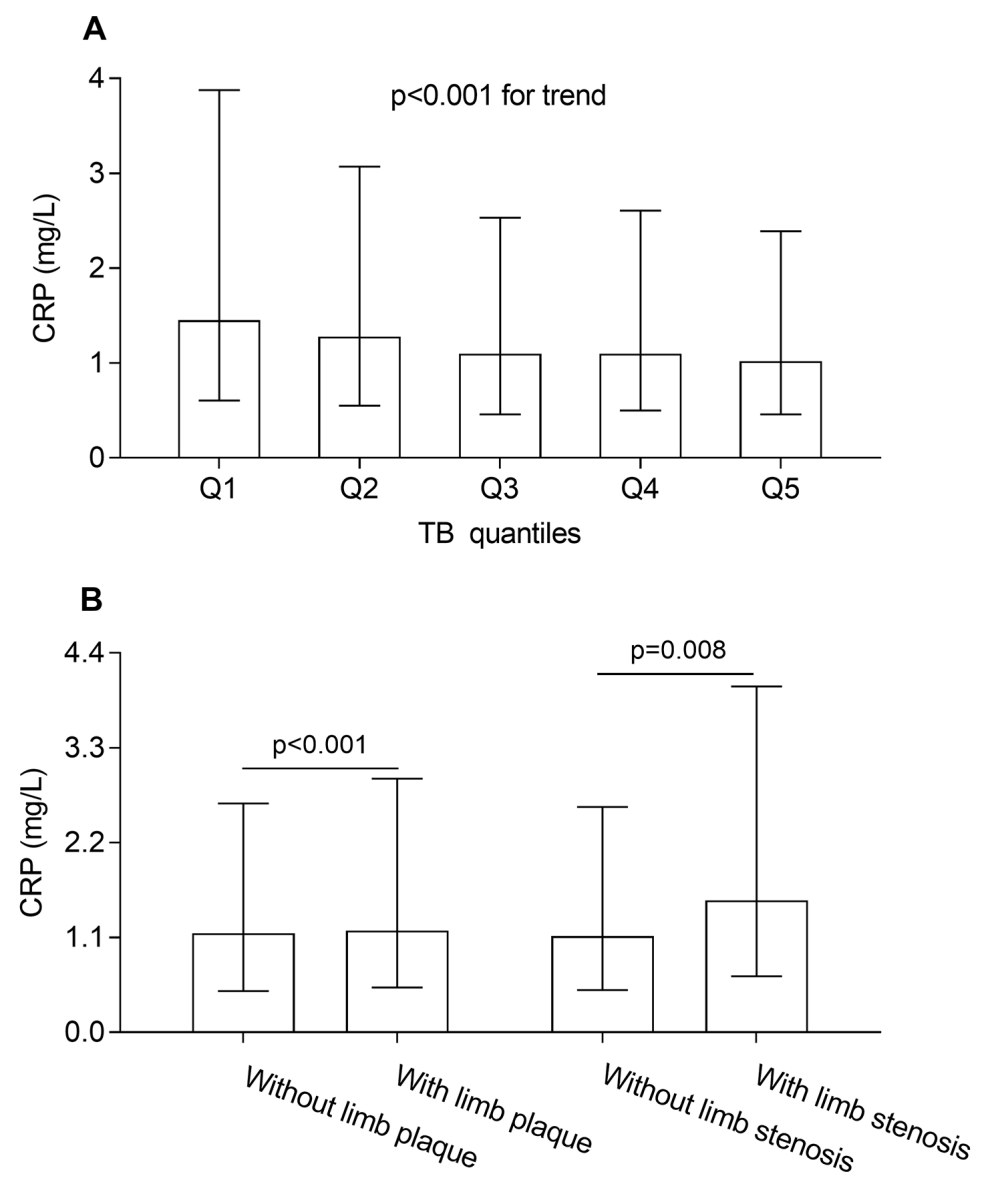
Association of serum bilirubin levels with CRP. (**A**) Comparison of CRP levels among the TB quintile groups. (**B**) Comparison of CRP levels between the subjects with and without lower limb plaque/stenosis after controlling for age, sex, and DD.

A Spearman’s correlation analysis revealed that serum TB (r = -0.107, *p* < 0.001), CB (r = -0.054, *p* < 0.001), and UCB (r = -0.103, *p* < 0.001) levels were all negatively correlated with CRP even after adjusting for age, sex, and DD, respectively. In addition, Fig. 2 shows the comparison of CRP in different groups. A significant decrease in CRP levels was found from the lowest to the highest TB quintile group (*p* < 0.001 for trend, Fig. 2A). The levels of CRP were dramatically increased in the patients with lower limb plaque in relative to those without lower limb plaque [1.18 (IQR 0.52–2.94) vs. 1.15 (IQR 0.48–2.66) mg/L, *p* < 0.001], and the same was found in lower limb stenosis [1.53 (IQR 0.65–4.01) vs. 1.12 (IQR 0.49–2.61) mg/L, *p* = 0.008] (Fig. 2B).

## Discussion

The present study conducted in a large sample of T2DM patients with a physiological concentration of serum bilirubin observed that serum TB level was negatively associated with lower limb plaque and stenosis, while serum UCB level was only correlated with a low occurrence of lower limb plaque and CB level was only associated with a low prevalence of lower limb stenosis, independent of other classical and nonclassical cardiovascular risk factors. Furthermore, low serum bilirubin levels were closely correlated with high CRP levels, which indicated that high-normal bilirubin may participate in attenuating atherosclerotic lesions via anti-inflammatory effect. To our knowledge, no previous studies have comprehensively linked the normal-range serum bilirubin levels to lower limb atherosclerosis in T2DM individuals at high cardiovascular risk.

Several previous studies have explored the associations of serum bilirubin with lower limb atherosclerotic disease with inconsistent conclusions, mainly referred to PAD [9, 25–29]. For example, the National Health and Nutrition Examination Survey found that a 0.1 mg/dL rise in bilirubin level was correlated with a 6% decrease in the incidence of PAD [25]. Nevertheless, a retrospective cross-sectional study by Nishimura et al. [28] noted that serum TB concentration was not related to the occurrence of PAD in T2DM patients. Furthermore, only one observational cohort study showed that serum levels of TB, D-BIL and I-BIL, within the normal range, were remarkably lower in PAD patients compared with healthy volunteers [30]. Consistent with this, we also found that the T2DM patients with lower limb stenosis exhibited significantly lower serum TB, CB and UCB levels even within normal limits compared with those without. These findings were further supported by the study from Lapenna and colleagues [31]. They observed that the levels of serum TB, CB and UCB were lower in the subjects with a carotid stenosis degree ≥ 90% compared with those with < 90% stenosis [31]. Meanwhile, we also observed that the patients with lower limb plaque started to exhibit a significantly lower serum UCB concentration, rather than TB and CB concentrations, compared with those without lower limb plaque, which suggested that UCB may have an earlier predictive ability for lower limb atherosclerosis than TB and CB. It could attribute to the fact that UCB has more effective and potent antioxidant and anti-lipoperoxidative properties than CB by virtue of its hydrophobicity [31–33].

Additionally, up to now, few studies have been made on the effect of serum bilirubin on lower extremity vascular lesions especially atherosclerosis in both non-diabetic and diabetic population. Our present investigation mainly focused on the association of serum bilirubin with lower limb atherosclerosis, a preclinical state of PAD, in T2DM patients with the physiological range of serum bilirubin. Our study revealed that 1SD increment in TB level within the normal range was associated with a 13% decreased risk of lower limb plaque and 16.5% in stenosis in T2DM patients after adjusting for multiple confounding factors. Of note, we found that high-normal UCB level was only negatively related to lower limb plaque while high-normal CB was only negatively related to lower limb stenosis. Similar with us, Hamur et al. [34] reported that TB level rather than direct bilirubin was a standalone predictor for subclinical atherosclerosis (carotid IMT ≥ 0.9 mm) in patients with prediabetes. Likewise, Muccini et al. [35] observed that increased total [adjusted OR (95%CI) 0.57 (0.36–0.90), *p* = 0.016] and indirect bilirubin [0.62 (0.40–0.97), *p* = 0.036] conferred lower risk of carotid lesions (defined as an CIMT ≥ 1.5 mm) in HIV-Infected patients with virological suppression, but direct bilirubin had no this effect. This could be explained by the fact that UCB properly and directly interacts with lipids owing to its hydrophobicity, while CB possesses the stronger hydrophilic properties, leading to inadequate hydrophobic interactions with biological lipids [33, 36].

TB has been earlier found to be inversely related to ultrasound-measured carotid and lower limb atherosclerosis in few, often small studies with non-selected population. Ishizaka et al. [37] first reported that the subjects with carotid plaque had remarkably lower serum bilirubin levels compared with those without carotid plaque, and an increase of 17.1 mmol/L in serum bilirubin concentration could result in an odds ratio of 0.37 for carotid plaque among 1741 individuals underwent general health screening tests. This team later verified this idea again in a larger correlation study involving 8,144 health check-up subjects [38]. Subsequently, similar results were found in hypertensive, elder, and prediabetic populations [34, 39, 40]. However, only one prior study evaluated the association of serum TB with the atherosclerosis of femoral arteries in 464 individuals with familial dyslipidemia, in which serum TB levels of 16 participants were beyond the normal range [14]. They found that a negative correlation between serum TB and femoral plaque thickness (β = -0.183; *p* = 0.030) only existed in the subjects with familial combined hyperlipidemia [14]. In the present study with a large sample of T2DM patients, we explored the role of serum bilirubin including TB, UCB and CB within the normal range on lower limb atherosclerosis and found that serum bilirubin levels were negatively and independently correlated with both lower limb plaque and stenosis in a dose-dependent way. Importantly, the most well-known determinants of atherosclerosis such as LDL-C and statin therapy were considered in our statistical analyses, which were often ignored by prior studies.

Although the exact mechanism underlying the impact of serum bilirubin within the physiological range on lower limb atherosclerosis is still unclear, the anti-inflammatory effect of bilirubin in limb atherosclerosis may partly explain this phenomenon, which was indicated by the alterations of serum CRP levels in our study. Several studies have reported that serum TB level was inversely correlated with CRP in overweight individuals and patients with coronary atherosclerosis [41, 42]. Aligned with them, we also found that serum TB, CB and UCB levels within the normal reference were negatively associated with CRP in patients with T2DM, which indicated that high-normal bilirubin may exhibit powerful anti-inflammatory activity against lower limb atherosclerosis.

There are some limitations in our study. The current study consisted of Chinese patients with T2DM. Therefore, the generalizability of our findings to other populations, races and ethnic minorities needs further verification. Additionally, based on the nature of this cross-sectional study, the present findings are unable to establish a causal relationship between serum bilirubin and lower limb atherosclerosis and thus further studies are needed to verify. Nonetheless, the present study was a real-world study, which was more conforming to clinical practice. Moreover, we added more information to the medical literature, supporting the role of bilirubin even within the physiological range on the prevention of atherosclerosis. However, further large-scale prospective studies are needed to address and clarify these issues.

## Conclusions

In conclusion, our current study made the first preliminary validation that lower-normal serum bilirubin was independently related to an increased risk of lower limb plaque and stenosis in T2DM patients. Our findings strongly supported and extended the notion that elevated bilirubin levels, even within the normal range, may exhibit an anti-inflammatory effect on the lower limb atherosclerotic process. Serum bilirubin could be used as a clinically simple and helpful indicator to assess the risk of lower limb atherosclerosis in T2DM patients.

## Electronic supplementary material

Below is the link to the electronic supplementary material.

Supplementary Material 1: Characteristics of serum bilirubin levels in the study subjects stratified by sex, age, and DD. (**A**) Comparison of the TB levels stratified by sex after adjusting for age and DD. (**B**) Comparison of the TB levels stratified by age after adjusting for sex and DD. (**C**) Comparison of the TB levels stratified by DD after adjusting for sex and age. (**D**) Comparison of the CB levels stratified by sex after adjusting for age and DD. (**E**) Comparison of the CB levels stratified by age after adjusting for sex and DD. (**F**) Comparison of the CB levels stratified by DD after adjusting for sex and age. (**G**) Comparison of the UCB levels stratified by sex after adjusting for age and DD. (**H**) Comparison of the UCB levels stratified by age after adjusting for sex and DD. (**I**) Comparison of the UCB levels stratified by DD after adjusting for sex and age

Supplementary Material 2: Characteristics of lower limb atherosclerosis in the subjects with T2DM. (**A**) Comparison of the prevalence of lower limb plaque stratified by sex after adjusting for age and DD. (**B**) Comparison of the prevalence of lower limb plaque stratified by age after adjusting for sex and DD. (**C**) Comparison of the prevalence of lower limb plaque stratified by DD after adjusting for age and sex. (**D**) Comparison of the prevalence of lower limb stenosis stratified by sex after adjusting for age and DD. (**E**) Comparison of the prevalence of lower limb stenosis stratified by age after adjusting for sex and DD. (**F**) Comparison of the prevalence of lower limb stenosis stratified by DD after adjusting for age and sex

## Data Availability

The datasets used and/or analysed during the current study are available from the corresponding author on reasonable request.

## Abbreviations

PAD: peripheral arterial disease
LEA: lower-extremity amputation
T2DM: type 2 diabetes mellitus
TB: total bilirubin
CB: conjugated bilirubin
D-BIL: direct bilirubin
UCB: unconjugated bilirubin
I-BIL: indirect bilirubin
BMI: Body mass index
WHR: waist-to-hip ratio
FPG: fasting plasma glucose
HbA1c: glycosylated hemoglobin A1c
2h PPG: 2-h postprandial plasma glucose
FCP: fasting C-peptide
2h PCP: 2-h postprandial C-peptide
Fins: fasting insulin
2hins: 2 h insulin
TTG: total triglycerides
TC: total cholesterol
HDL-C: high-density lipoprotein cholesterol
LDL-C: low-density lipoprotein cholesterol
Lp(a): Lipoprotein a
ALT: alanine aminotransferase
AST: aspartate aminotransferase
γ-GT: γ-glutamyl transpeptidase
Cr: creatinine
SUA: serum uric acid
CRP: C-reactive protein
UAE: urinary albumin excretion
HOMA-IR: homeostasis model assessment index of insulin resistance
HOMA2-IR: homeostasis model assessment for insulin resistance
eGFR: estimated glomerular filtration rate
IMT: intima-media thickness
FIMT: femoral IMT
IQR: interquartile range
DD: duration of diabetes
LLDs: lipid-lowering drugs
APAs: anti-platelet agents
IIAs: insulin analogues
OR: odds ratio
CI: confidence interval
DF: diabetic foot.
