# Supplementary figures and images for: High-normal serum bilirubin decreased the risk of lower limb atherosclerosis in type 2 diabetes: a real-world study
Source: Diabetol Metab Syndr. 2023 May 19;15:105. doi: 10.1186/s13098-023-01088-9 (PMC10197852; doi:10.1186/s13098-023-01088-9)

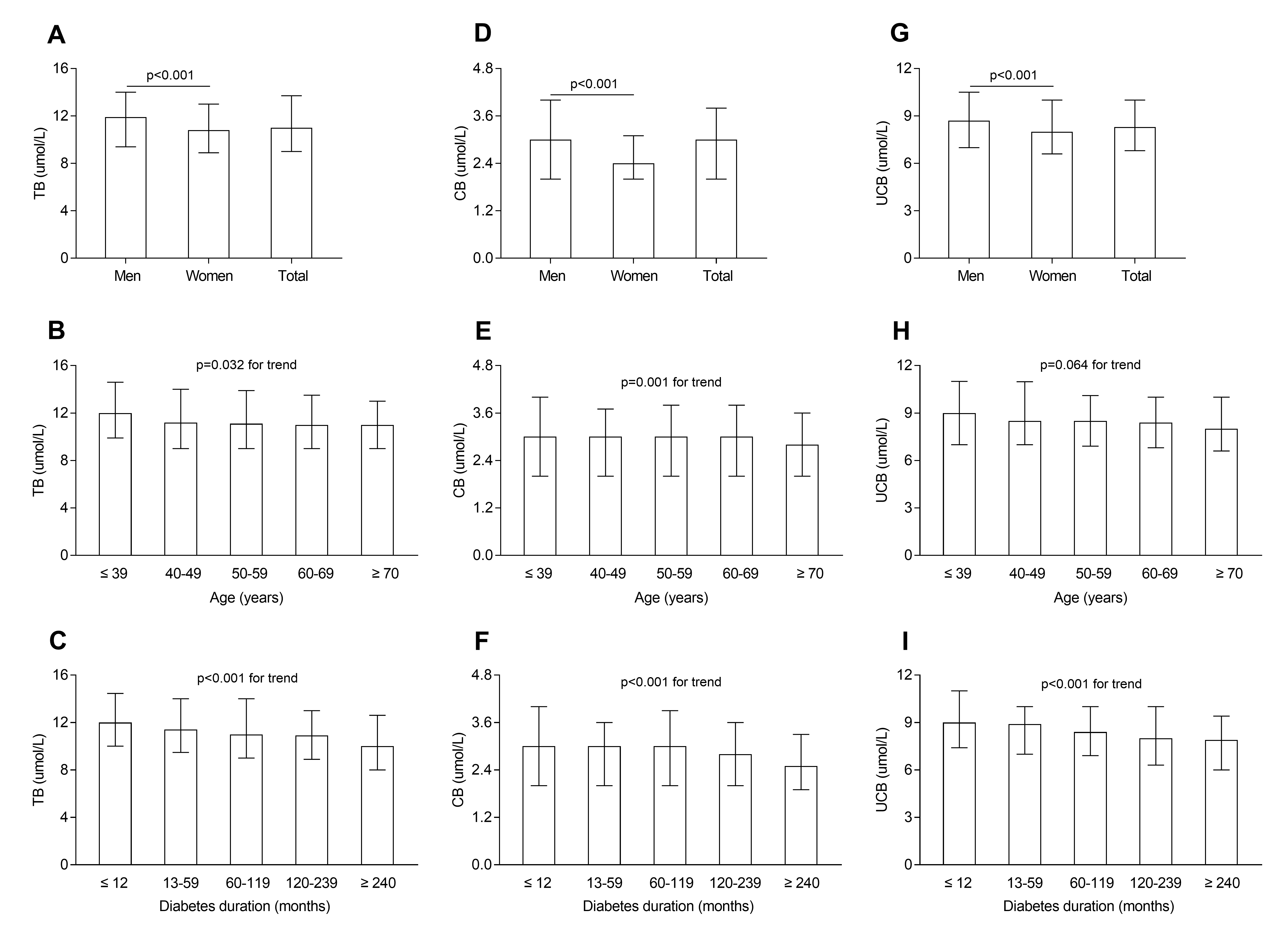

Supplement: Supplementary file 1 — Supplementary Material 1: Characteristics of serum bilirubin levels in the study subjects stratified by sex, age, and DD. (A) Comparison of the TB levels stratified by sex after adjusting for age and DD. (B) Comparison of the TB levels stratified by age after adjusting for sex and DD. (C) Comparison of the TB levels stratified by DD after adjusting for sex and age. (D) Comparison of the CB levels stratified by sex after adjusting for age and DD. (E) Comparison of the CB levels stratified by age after adjusting for sex and DD. (F) Comparison of the CB levels stratified by DD after adjusting for sex and age. (G) Comparison of the UCB levels stratified by sex after adjusting for age and DD. (H) Comparison of the UCB levels stratified by age after adjusting for sex and DD. (I) Comparison of the UCB levels stratified by DD after adjusting for sex and age [file 13098_2023_1088_MOESM1_ESM.tif]

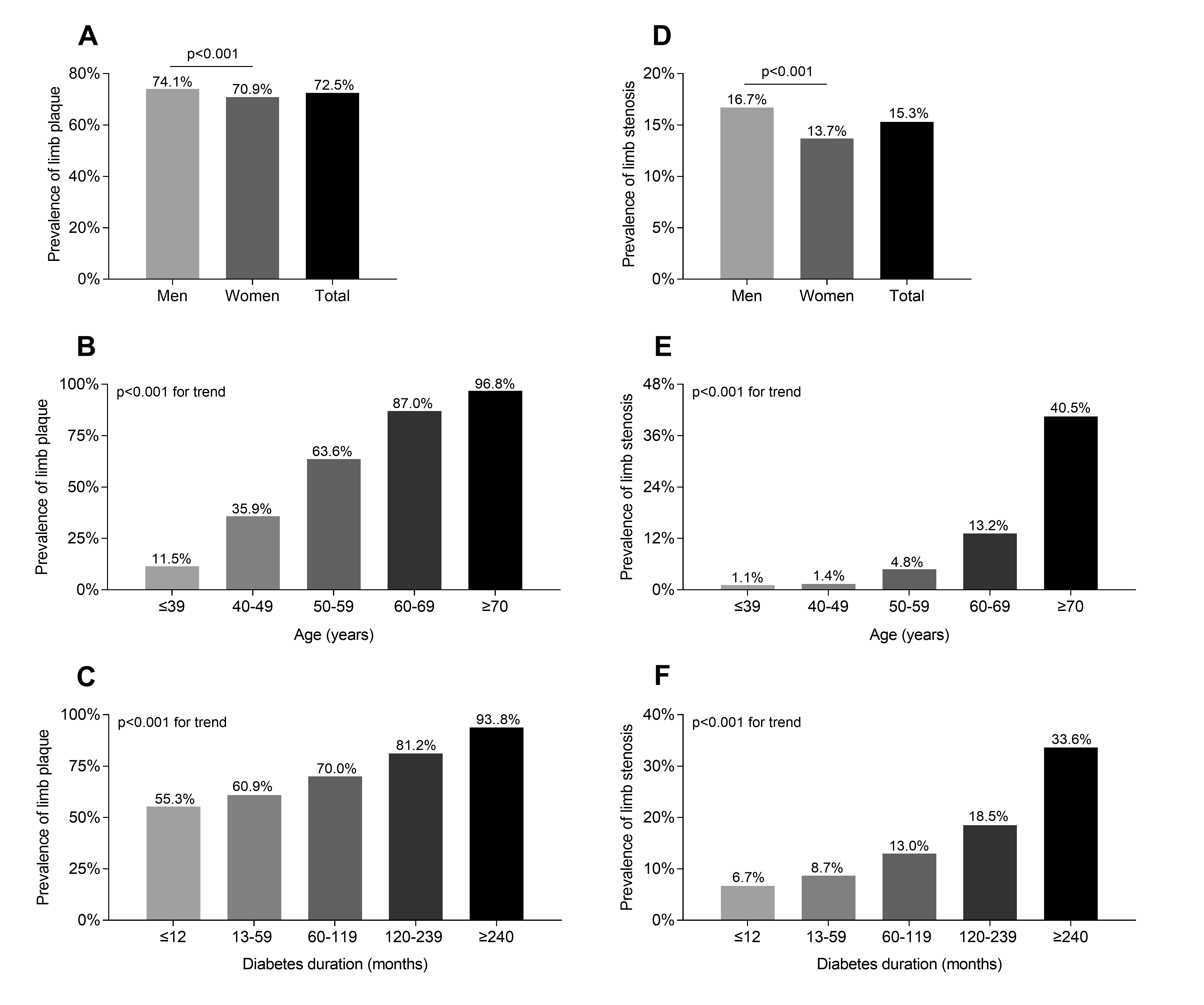

Supplement: Supplementary file 2 — Supplementary Material 2: Characteristics of lower limb atherosclerosis in the subjects with T2DM. (A) Comparison of the prevalence of lower limb plaque stratified by sex after adjusting for age and DD. (B) Comparison of the prevalence of lower limb plaque stratified by age after adjusting for sex and DD. (C) Comparison of the prevalence of lower limb plaque stratified by DD after adjusting for age and sex. (D) Comparison of the prevalence of lower limb stenosis stratified by sex after adjusting for age and DD. (E) Comparison of the prevalence of lower limb stenosis stratified by age after adjusting for sex and DD. (F) Comparison of the prevalence of lower limb stenosis stratified by DD after adjusting for age and sex [file 13098_2023_1088_MOESM2_ESM.tif]
